# Indents

**Published:** 1903-10-15

**Authors:** 


### INDENTS.

☞ **Brief Articles of Technical or Scientific Value will receive notice and comment. Contributions invited.**

**Electricity in the Dental Office.** Will it pay to put electricity in my office? Yes, by all means; you can not afford to be without it. Make friends with electricity, study it and familiarize yourself with its characteristics. It is unapproachable in promptness, being ever ready to serve you, by lighting your way, running your engine and lathe, heating your furnace and therapeutic appliances. It rings your office bell, becoming the first greeting of an approaching patient or friend. It will run your automatic mallet or automobile. It keeps you and your patients cool in the hot summer, and carries your voice instantaneously to distant parts of the city or the world if need be.

The sun is the great source of electricity; have plenty of it in your office, no matter in what form. Have it in your life as well as your soul.—A. M. Hewett, in *Dental Review.*

**Antidolorin For Minor Surgery.** This will be its main use with most dentists and if used skillfully a painless extraction can be accomplished. Isolate the gum over tooth to be extracted (by means of absorbents), spray on antidolorin until gum becomes perfectly white, and after tissues regain the normal color make extraction; it is better to anesthetize gums on both sides of tooth wherever practicable. In the lower jaw, if possible, anesthetize tissues about the inferior dental foramen, so as to get a better anesthesia of the nerves making their exit there; by this method good results are obtained.

It can be sprayed into teeth which are very sensitive and although the first application will cause pain it will remove the sensitiveness from a pulp long enough to allow you to make undercuts for a filling. In removing an exposed pulp spray into cavity until all pain ceases, then remove pulp. There is absolutely no gangrene or after pain from its use.

**Formaldehyde for Pulp-Canal Cleansing.** MM. Andre and Marion recommend the following formula:

Formic aldehyde, 40 parts;
Essence of geranium, 20 parts;
Alcohol, eighty percent, 40 parts.

They claim that the alcohol, by uniting with and dissolving the fatty matter always present in a foul pulp-canal, assists very materially the coagulation of the albuminous contents by the formaldehyde. They also claim that until the pulp-canal is thoroughly clean formaldehyde is not a sterilizer; therefore its application should be continued after the cleanliness of the canal has been proved by the usual tests. The above formula has resulted from a long series of carefully conducted experiments, undertaken with the purpose of utilizing the valuable properties of this agent in treating putrescent conditions of the pulp-canal and its surroundings safely, efficiently, and with the least discomfort to the patient.—*British Journal of Dental Science*, June 1, 1900, page 481.

**Chinese Dentistry.** Dr. R. S. Ivy, of Shanghai, China, sends the following translation of a Chinese quack dental advertisement: "Since I was small I have studied dentistry, I graduated at a great college and have got a diploma. Since then I have practiced in China many years. My fame exists in all the provinces. Every year I visit the North. All kinds of troubles with the teeth I will cure. I will fill up the holes made by worms.

"Tooth-ache arises from wind, fire and worms, which eat up the teeth. When you eat the pressure of food causes pain. I can repair such places and perpetual relief will result. Whether you have only lost a few or all of your teeth, I can make you a whole new set on which you can chew the toughest objects. Fillings are made of gold, silver and pewter. Plates are made of gold skin or elephant skin. I can do this. This is true. Prices can be arranged so as to suit every one. Please come early. Signed, Ho-Ching-Toong.''—*Digest.*

**Chloroform Liniment for Dental Use.**—

Chloroform, ℥ii,

Ether, ℥ii;

Alcohol, ℥i;

Gum-camphor, ℥i.

This is a very valuable preparation, and should be kept handy in every dental office. A lock of cotton, liberal in size, saturated with it and placed on the gum over a painful tooth, usually gives prompt relief. It is especially useful in painful conditions following a arsenical application, pulp-canal filling, the insertion of a crown or bridge, and the after-pain of tooth-extraction. It usually mitigates very much the pain attending an alveolar abscess, and is a comfort to the patient if used before beginning the preparation of a root for crowing. Its efficiency is increased by adding a small portion of chloretone immediately before making the application. With the addition of a very little formaldehyde and chloretone or cocain, it may be used to anesthetize a pulp preparatory to its extirpation. When applied to the gums the first effect is usually a sharp and rather severe smarting, which passes off in a minute or two.—W. H. T., in *International Journal.*

**Mouth Breathing and Respiratory Obstructions.** I know a man forty-eight to fifty years old who has never been a mouth-breather, yet he has one of the most pronounced deviated septums that I have seen, the nasal passages are filled with adenoid growths and spurs. A child was a mouth-breather very early in life. I saw the little one when she was perhaps four years old, she is now fourteen, but only in the last two years she has developed a most alarming case of adenoid growths, spurs, and some deviation and a closure of the septum—a closing of the throat by an enlargement of the tonsils which has come only in the last two years or after she was twelve years old. It is an open question, in my mind at least, as to which of these processes come first—thumbsucking, which tends to enlarge or throw out the upper incisors, or perhaps a pressure on the lower jaw producing a lack of development—I am not prepared at all to say. No such children should come into our offices without the parents being informed of their condition, and be recommended to a rhinologist who is able to take care of this kind of cases.

This child was one of the most beautiful children, but in the last two years there has been a real protrusion of the upper jaw, and there may be the recession in the lower jaw. The child has also degenerated mentally.—Dr. Hungerford in *Western Journal.*

**Care of the Hands.** It is not often that attention is called in societies to the necessity of more care by the dentist, in regard to the hands and instruments. A case of infection that I recently had to trace up has proved to my mind that we all ought to be more careful than we are. The case was that of a young, healthy woman about twenty-five years of age, who was in the country, not exposed to infection of any kind. She went to a dentist and had some teeth filled. This dentist was a consumptive in pretty bad shape, and a few days after he finished his work her mouth became very sore and her teeth loose, and three weeks afterwards she had a hard cough, and rapidly developed all symptoms of consumption. Examination of the sputum and the lungs showed tuberculosis bacilla in the saliva, and a consolidation in the lung. It is generally difficult to trace the case to the man who is responsible for it; but to my mind there are many cases where infection is given by the dentist through his hands or instruments. I think the dentist's hands are often responsible. They do not clean them properly. I dare say among the members of this society you will not find a bichloride solution in one office out of fifty; and yet dentists handle consumptive cases, and go from one to the other, just washing their hands with soap and water. It is not fair to the patient, and I think it ought to be brought more fully before the dentists. If you use alcohol and glycerin between the washings, you can have your hands soft and nice at the end of the day.—Dr. Russell, Brooklyn, *Items of Interest.*

**Antidolorin As a General Anesthetic.** It has been, as yet, very little used in dentistry. If it were better known in this field, it would be oftener used, mainly on account of the simplicity and cheapness of the apparatus required and the absolute safety with which it can be handled on all manner of patients. In this respect it ranks favorably with nitrous oxid. Its field of usefulness is wherever nitrous oxide can't be administered. The apparatus which has been found the best is at the same time the simplest and consists merely of a mask. The mask is placed over patient's mouth and nose and a stream of ethyl chlorid is sent into the receiving tube, intermittently, at each inhalation of the patient. The tube should be held about eight inches distant from the inhaler. When a hoar frost gathers over the gauze the stream should be stopped until this has evaporated, as otherwise the antidolorin is lost and not inhaled. The average amount for a complete anesthesia is about ten grammes. No cyanosis follows, the muscles are in a relaxed condition and the patient recovers very quickly without the least disagreeable after-effects. In operations about the mouth a prop is advisable although not absolutely necessary.

To a practitioner who has to see patients at their homes and administer an anesthesia for minor surgical operations it is the most portable and safest apparatus.

Patients can be kept under its influence safely much longer than nitrous oxide and for a general anesthetic it can be used by the dentist for the same operations wherever it is used locally.—Dr. M. Greene.

**Dental Vestibules.** The human body is often spoken of as a "temple" and also as "the house we live in." This being the case, the mouth is the vestibule, as it is the place of entrance.

There has been just as much change in our status as dentists during the last thirty years as there has been in the vestibules of our houses. In former days, these vestibules were far from being inviting places. They were narrow, without ornament, dark, cold, and badly furnished, serving merely as places of entrance and places of deposit for outer garments, especially those made from products of the rubber tree.

So, formerly, the mouth was a place of small consideration in the human house. It was also often narrow from contracted arches and dark from decayed teeth. It was scantily furnished and the furniture in a bad state of repair. It was also largely a place of deposit and ornaments made from the products of the rubber tree were apt to be more conspicuous than ivories.

At that time, when the dentist was admitted to this vestibule it was very grudgingly, his stay was short and as he was badly, or inadequately paid, he generally retired with some of the remaining ivories.

Now we have reception halls in our houses, which are large and inviting, well furnished and in which ornaments are not lacking.

To-day, when a dentist is invited to enter the vestibule of the human body, he generally finds it a more inviting place than formerly, and, as the owner takes much more pride in its furnishing and preservation, the work required in keeping it in order is not so laborious.—Dr. McFarlane in *Review*.

**Literature in Dental Offices.** Without fear of successful contradiction, we make the statement that not one dentist in fifty even makes a pretense of keeping current literature on the table in his reception room. Most dentists apparently consider their full duty discharged if they have a few tattered and torn magazines of ancient vintage displayed for the edification of their patients. A visit to the dental office is not a cheerful prospect for the average individual, and some have suggested that the reason why dentists keep such unattractive reading matter on their tables is because, after a few moments' perusal of it, the patient is glad to escape anywhere from it—even into the operating chair. Seriously speaking, however, dentists would find that the outlay of say one dollar per month for the standard current magazines would repay them several times over. The average patient, man or woman, dreads the work which is to be done, and the longer he or she must wait in the reception room the greater the dread and uneasiness. A few moments' perusal of the standard popular literature of the day would take their minds off themselves and the coming operation, and they would come into the office in a more cheerful frame of mind, and consequently in much better condition for the operator. Anything that can divert the mind into pleasant channels while waiting for the dentist is a positive godsend, and your patients will appreciate a little attention along this line. Another very good reason for keeping late standard literature on the reception room table is that patients, especially new ones, are apt to judge by externals, and you cannot blame them for thinking that if a man is two or three years behind in literature he would also be in his work. You would not ask your patients to sit in uncomfortable, broken-down chairs, so why ask them to be content with aged, dilapidated and unattractive reading matter?—*Digest.*

